# Monoclonal antibody A7-superparamagnetic iron oxide as contrast agent of MR imaging of rectal carcinoma

**DOI:** 10.1038/sj.bjc.6602668

**Published:** 2005-06-21

**Authors:** A Toma, E Otsuji, Y Kuriu, K Okamoto, D Ichikawa, A Hagiwara, H Ito, T Nishimura, H Yamagishi

**Affiliations:** 1 Department of Surgery, Kyoto Prefectural University of Medicine, 465 Kawaramachi Hirokoji Kamigyo-ku, Kyoto 602-8566, Japan; 2 Department of Radiology, Kyoto Prefectural University of Medicine, 465 Kawaramachi Hirokoji Kamigyo-ku, Kyoto 602-8566, Japan

**Keywords:** recurrence of rectal carcinoma, magnetic resonance imaging, contrast agent, monoclonal antibody, superparamagnetic iron oxide

## Abstract

Superparamagnetic iron oxide (SPIO)-based colloid has been used clinically as a tissue-specific magnetic resonance contrast agent. We coupled monoclonal antibody A7 (Mab A7), which reacts specifically with human colorectal carcinoma, to Ferumoxides (SPIO) and examined the accumulation of this conjugate in xenografted tumours in nude mice. We examined *in vitro* immunoreactivity of Mab A7 coupled to Ferumoxides and its *in vivo* distribution in nude mice with human colorectal carcinoma. Magnetic resonance imaging of tumour-bearing nude mice was performed 72 h after injection of A7-Ferumoxides. A7-Ferumoxides retained binding activities that were nearly identical to intact Mab A7. More of the radiolabelled A7-Ferumoxides accumulated in the tumour than normal mouse IgG-Ferumoxides from 12 h onwards after injection (*P*<0.05). Both A7-Ferumoxides and normal mouse IgG-Ferumoxides disappeared from blood linearly over time. The accumulation levels in normal tissue decreased linearly over time but were lower than levels in tumours from 6 h. In magnetic resonance T2-weighted imaging of the tumour-bearing nude mice, signal intensity was reduced at the margin of the tumour by injection of A7-Ferumoxides. Mab A7 coupled to Ferumoxides is potentially suitable as a magnetic resonance contrast agent for detecting local recurrence of rectal carcinoma.

As chemotherapy, radiotherapy, and thermotherapy adjuncts to surgical treatment are performed for rectal carcinoma, the survival rate has improved in recent years (Hildebrandt *et al*, 2000; Adell *et al*, 2001; Frykholm *et al*, 2001). Total mesorectal excision for rectal cancer has reduced the rate of local recurrence and raised overall survival. Yet, local recurrence, which is the most common pattern in rectal carcinoma, occurs in approximately 8–15% of patients even after curative surgery (Goldberg and Nicholls, 1995; Wibe *et al*, 2003). Moreover, a diagnostic problem is represented by the difficult differentiation of local recurrence from postoperative fibrosis by either computed tomography (CT) or magnetic resonance (MR) imaging (Balzer *et al*, 2003). The development of contrast agents which accumulate in rectal carcinoma might eventually improve its prognosis.

Although MR imaging currently is considered the most accurate method for diagnosing local recurrence, unenhanced and gadolinium-enhanced MR imaging are not sensitive enough for differentiation between local recurrence and other tissues. Magnetic resonance imaging techniques have been proposed, including the use of ferromagnetic particles (Fe_3_O_4_) as MR contrast agents (Mendonca-Diasand and Lauterbur, 1986; Renshaw *et al*, 1986a,  b). Superparamagnetic iron oxide (SPIO)-based colloid has been investigated as an MR contrast agent (Weissleder *et al*, 1987a,  b). Ferumoxides, one SPIO-based colloid, has been used clinically as a tissue-specific MR contrast agent in order to distinguish liver tumours (Clement *et al*, 1991). No MR contrast agent has been shown to detect the local recurrence of rectal carcinoma.

We have previously reported that monoclonal antibody A7 (Mab A7), produced from human colonic carcinoma, reacts immunohistochemically with colorectal carcinomas with high sensitivity (Otsuji *et al*, 1992; Takahashi *et al*, 1993). In this study, we coupled Mab A7 to Ferumoxides in order to examine whether the complex would be useful as an MR contrast agent for local recurrence of rectal carcinoma.

## MATERIALS AND METHODS

### Cell line

WiDr, the human colonic carcinoma cell line, was used in this study. WiDr cells were incubated in RPM1 1640 media supplemented with 10% foetal bovine serum (FBS) in an atmosphere of 5% CO_2_ and air at 37°C.

### Tumour xenograft

Cultured WiDr cells were harvested by ethylenediamine tetraacetic acid (EDTA) treatment, washed, and resuspended in PBS. Approximately 10^7^ viable cells were injected subcutaneously into the backs of athymic 8-week-old nude mice (BALB/C-nu/nu, male, mean body weight: approximately 25 g) (Shimizu, Shizuoka, Japan). A tumour mass was detected in all mice injected with WiDr cells 14 days after inoculation.

### Monoclonal antibody A7

Monoclonal antibody A7 was produced from spleen cells of a mouse immunised against human colonic adenocarcinoma, Colon 6 (Kotanagi *et al*, 1986). Monoclonal antibody A7 belongs to the IgG_1_ subclass and recognises a 42 000 Da glycoprotein on the cell surface. It has been reported to react with more than 70% of human colorectal carcinoma cell lines (Takahashi *et al*, 1993), but it does not react immunohistochemically with normal tissues. Normal mouse IgG was purchased from Sigma-Aldrich (St Louis, MO, USA).

### Preparation of radiolabelled Mab

Radiolabelling of Mab A7 with ^125^I (Amersham Japan, Tokyo) was performed by the chloramine-T method (Hunter and Greenwood, 1962). The iodinated Mab A7 was separated from excess reactants by passage through a Sephadex G-25 column. Monoclonal antibody A7 was labelled with ^125^I to specific activities of 5.0 *μ*Ci *μ*g^−1^. As a control, normal mouse IgG also was labelled with ^125^I by a similar method.

### Preparation of SPIO

Ferumoxides, a SPIO - based colloid, was obtained from Advanced Magnetics Co. (Cambridge, MA, USA). Ferumoxides is a colloid of SPIO (FeO and Fe_2_O_3_) supported with dextran T-10, and the mean diameter of particles is approximately 100 nm. It contained 11.2 mg Fe ml^−1^.

### Mab A7 coupled to Ferumoxides

Monoclonal antibody A7 was coupled with the dextran of Ferumoxides using a modification of the sodium periodate method (Molday and Mackenzie, 1982). In all, 1 ml of Ferumoxides was added to 10 mM sodium periodate. The dextran of Ferumoxides was partially oxidised. The reaction was carried out for 60 min at room temperature with stirring, the oxidised Ferumoxides was separated from residual periodate by passage through a Sephadex G-25 column. Then 3 mg of Mab A7 was added to the solution of oxidised Ferumoxides. The aldehyde functions of oxidised dextran react with the amino sites of Mab A7 to form Schiff bases. The conjugation reaction was carried out for 24 h at room temperature with stirring, 0.5 ml of 1% sodium borohydride solution was then added in order to reduce the product to secondary amine linkages. After 15 min, the reaction was terminated by dialysing against 0.1 M sodium phosphate buffer. Ferumoxides coupled to antibody was separated from unconjugated antibody by centrifugation for 15 min at 15 000 rpm. The supernatant was discarded, and the pellet containing the A7-Ferumoxides complex was resuspended. Similarly, Ferumoxides was coupled to normal mouse IgG as a control. Approximately 20% of the initially added antibody was coupled with Ferumoxides.

### Stability of A7-Ferumoxides in human serum

^125^I-labelled A7-Ferumoxides (0.5 mg) was added to volunteer human serum (0.5 ml), and the serum was allowed to clot in humidified CO_2_ (37°C, 5% CO_2_ atmosphere). At 24, 72, and 120 h after incubation, ^125^I-labelled A7-Ferumoxides was separated from unconjugated antibody by centrifugation for 15 min at 15 000 rpm. Radioactivities of 100 *μ*1 of each supernatant were counted with a gamma scintillation counter (COBRA 5002/5003, Packard, CO, USA) and were compared with the count of the supernatant of the sample prior to incubation.

### *In vitro* binding activities of A7-Ferumoxides to WiDr cells

The binding activities of A7-Ferumoxides were examined by a competitive inhibition radioimmunoassay in WiDr cells. Aliquots of WiDr cells (10^6^) were incubated with a fixed quantity of ^125^I-labelled A7 (10^5^ cpm) in the presence of serially four-fold diluted A7-Ferumoxides or intact A7 at 37°C for 60 min. The concentration of A7-Ferumoxides and intact A7 ranged from 10.0 × 4^−6^ to 10.0 *μ*g ml^−1^. As a control, normal mouse IgG-Ferumoxides was used instead of A7-Ferumoxides or intact A7. After incubation, the cells were washed three times with PBS. Radioactivity of the cell pellets was counted with a gamma scintillation counter (COBRA 5002/5003, Packard), and the percent inhibition was calculated compared to the results of only ^l25^I-labelled A7.

### Biodistribution of ^l25^I-labelled A7-Ferumoxides in nude mice bearing human colorectal carcinoma xenografts

The biodistribution of ^125^I-labelled A7-Ferumoxides was examined in nude mice bearing WiDr tumours. At 14 days after inoculation, 48 tumour-bearing nude mice were divided into two groups: 24 mice in one group were injected intravenously with 1.0 *μ*Ci of ^125^I-labelled A7-Ferumoxides via the tail vein, 24 mice in another group were injected with 1.0 *μ*Ci of ^125^I-labelled normal mouse IgG-Ferumoxides. Three mice from each group were killed and dissected at 2, 6, 12, 24, 48, 72, 96 and 120 h after injection. After dissection, the tumours, blood, and normal organs (lung, heart, liver, spleen, pancreas, stomach, colon, and kidney) were weighed. The mean weight of the tumours was 178 mg. The radioactivity in each tissue was counted with a gamma scintillation counter. The results are expressed as the percentage of the injected dose g^−1^ (%ID g^−1^). Furthermore, to compare the kinetics of the two probes in the tumour, blood, and normal tissues, the tissue/blood ratios of the radioactivity per unit weight were calculated from these data. Student's *t*-test was used to determine statistically significant differences which were set at *P*<0.05. Care of the animals and the experimental procedures were carried out in accordance with the guideline of the United Kingdom Co-ordinating Committee on Cancer Research (UKCCCR, 1998).

### MR imaging of nude mice bearing human colorectal carcinoma xenografts

A WiDr tumour-bearing nude mouse was studied under pentobarbital anaesthesia 72 h after injection of A7-Ferumoxides. All MR images were obtained with a 1.5 T clinical scanner with a small field of view (FOV) coil (Eclipse 1.5 T, Marconi Medical systems, UK). These images were obtained with a gradient echo sequence, so-called ‘T2^*^’. We used T2^*^ sequences to visualise the uptake of SPIO because the T2^*^ sequence was the most sensitive at visualising the magnetic susceptibility. A tumour-bearing nude mouse injected with IgG-ferumoxides was imaged at the same time.

## RESULTS

### Stability of A7-Ferumoxides in human serum

Radioactivities of each supernatant at 24, 72, and 120 h after incubation of ^125^I-labelled A7-Ferumoxides with human serum were almost identical to that of background and to supernatant before incubation.

### *In vitro* binding activities of A7-Ferumoxides to WiDr cells

**Figure 1:**
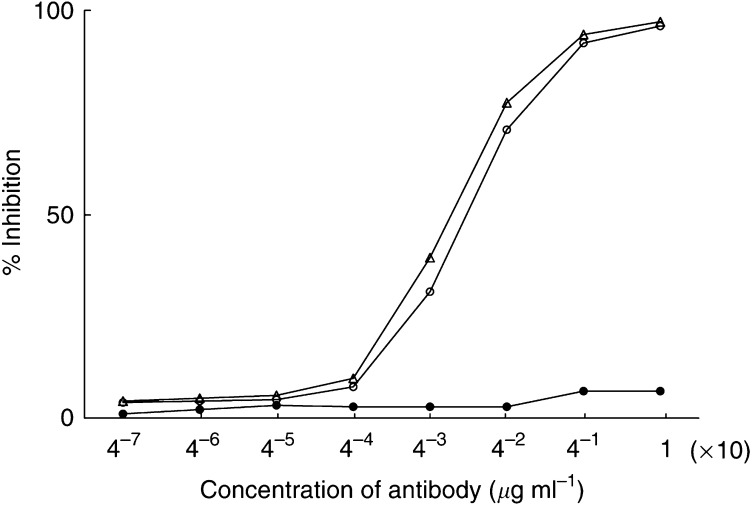
The binding activities of A7-Ferumoxides were compared with those of intact A7 by competitive radioimmunoassay in WiDr cells. A7-Femmoxides retained binding activities nearly identical to intact A7. Normal mouse IgG-Ferumoxides had no antigen-binding activity in WiDr cells. ○, A7-Ferumoxides; ▵, intact A7; •, normal mouse IgG-Ferumoxides.

The binding activities of A7-Ferumoxides were compared with those of intact Mab A7 by a competitive inhibition radioimmunoassay in WiDr cells. Mab A7-Ferumoxides retained binding activities which were nearly identical to intact Mab A7 (Figure 1). Normal mouse IgG-Ferumoxides did not react with WiDr cells.

### Biodistribution of ^l25^I-labelled A7-Ferumoxides in nude mice bearing human colorectal carcinoma xenografts

**Figure 2:**
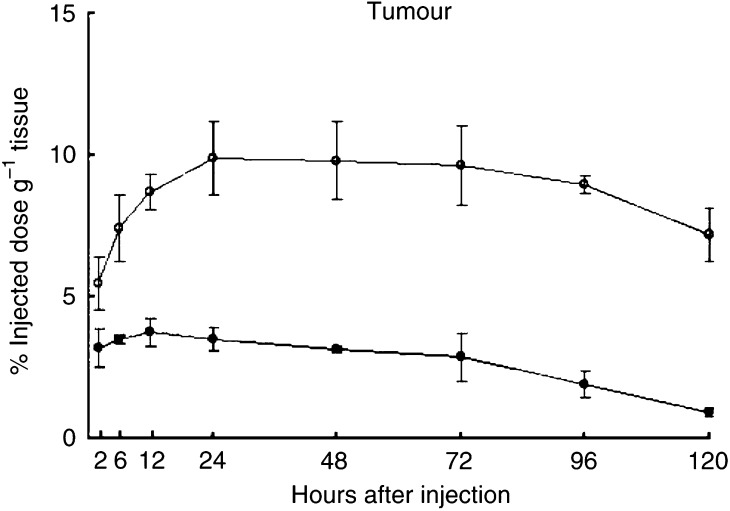
The accumulation of ^125^I-labelled A7-Ferumoxides and ^125^I-labelled normal mouse IgG-Ferumoxides in WiDr tumours of mice after intravenous injection. A significantly larger amount of ^125^I-labelled A7-Ferumoxides accumulated in the tumour than ^125^I-labelled normal mouse IgG-Ferumoxides from 12 to 120 h after injection (P<0.05). The tumour accumulation of ^125^I-labelled A7-Ferumoxides increased up to 48 h and then decreased slowly. ○, A7-Ferumoxides; •, normal mouse IgG-Ferumoxides; points, means; bars, s.d.

**Figure 3:**
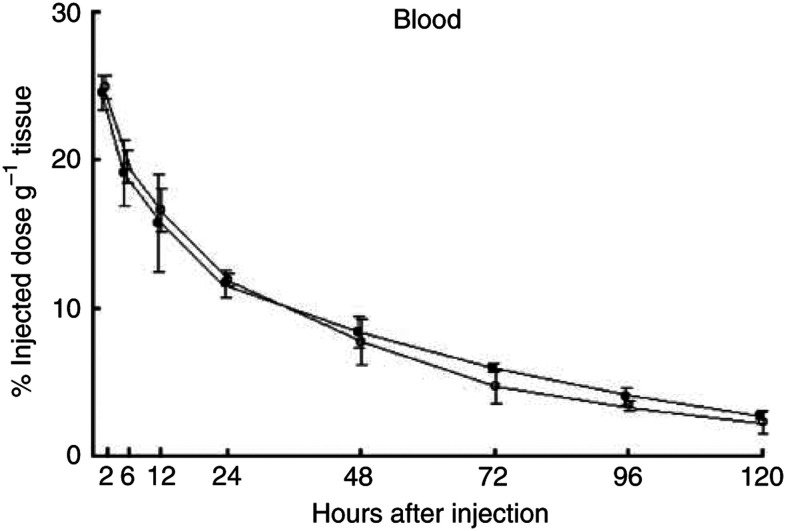
Blood concentrations of ^125^I-labelled A7-Ferumoxides and ^125^I-labelled normal mouse IgG-Ferumoxides in mice that received an intravenous injection. ^125^I-labelled A7-Ferumoxides and ^125^I-labelled normal mouse IgG-Ferumoxides disappeared from blood linearly over time, with similar clearance curves. ○, A7-Ferumoxides; •, normal mouse IgG-Ferumoxides; points, means; bars, s.d.

**Figure 4:**
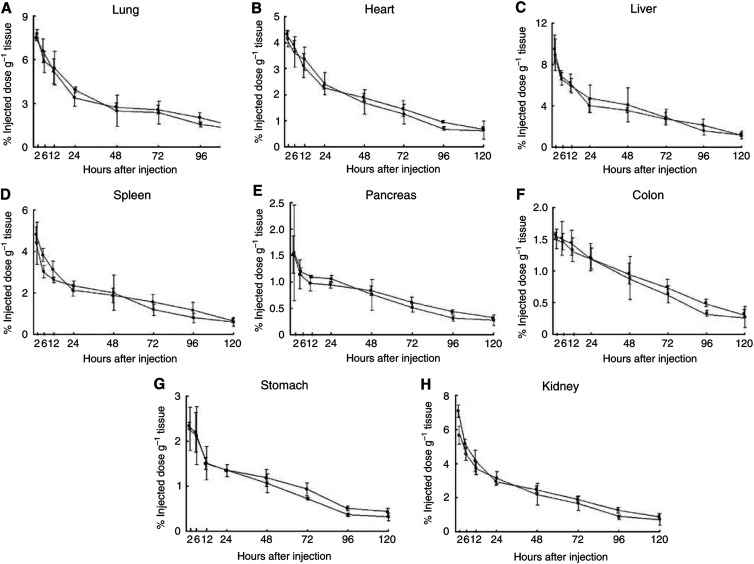
**A** to **H**. The accumulation of ^125^I-labelled A7-Ferumoxides and ^125^I-labelled normal mouse IgG-Ferumoxides in normal tissues of mice after intravenous injection. The accumulation of ^125^I-labelled A7-Ferumoxides and ^125^I-labelled normal mouse IgG-Ferumoxides was similar in normal tissues. ○, A7-Ferumoxides; •, normal mouse IgG-Ferumoxides; points, means; bars, s.d.

**Figure 5:**
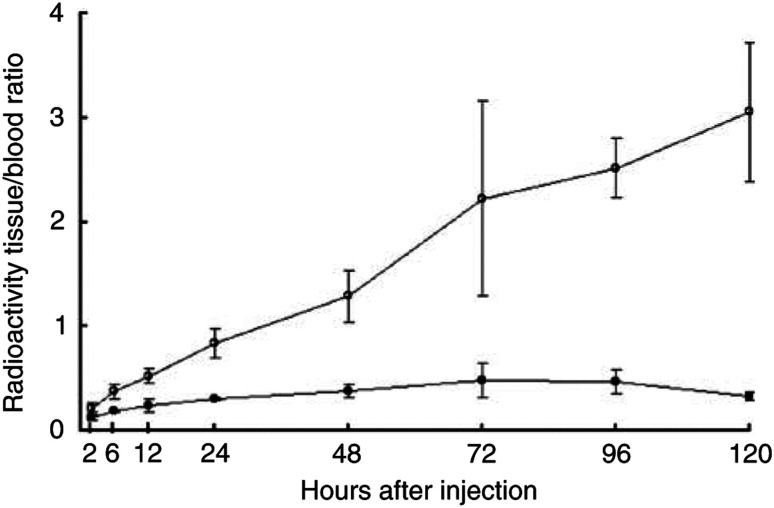
Tumour/blood radioactivity ratio of ^125^I-labelled A7-Ferumoxides and ^125^I-labelled normal mouse IgG-Ferumoxides in mice after intravenous injection. The tumour/blood radioactivity ratio of the ^125^I-labelled A7-Ferumoxides increased rapidly with time. ○, A7-Ferumoxides; •, normal mouse IgG-Ferumoxides; points, means; bars, s.d.

Significantly larger amounts of the ^125^I-labelled A7-Ferumoxides accumulated in the tumour than ^125^I-labelled normal mouse IgG-Ferumoxides from 12 to 120 h after injection (*P*<0.05). (Figure 2). The tumour accumulation level of ^125^I-labelled A7-Ferumoxides increased gradually, and radioactivity reached 9.87±2.96% ID g^−1^ 24 h after injection and then decreased slowly. By contrast, the tumour accumulation level of ^125^I-labelled normal mouse IgG-Ferumoxides decreased after radioactivity reached only 3.76±0.48% ID g^−1^ at 12 h after injection. ^125^I-labelled A7-Ferumoxides and ^125^I-labelled normal mouse IgG-Ferumoxides disappeared from blood linearly over time with similar clearance curves (Figure 3). As for all resected normal tissues, the accumulation levels of ^125^I-labelled A7-Ferumoxides decreased linearly over time and were lower than those for tumours from 6 h onwards after injection (Figure 4A–H). Accumulations of ^125^I-labelled A7-Ferumoxides and ^125^I-labelled normal mouse IgG-Ferumoxides were similar in normal tissues. To examine the specific localisation of ^125^I-labelled A7-Ferumoxides and ^125^I-labelled normal mouse IgG-Ferumoxides in tumours, the ratio of radioactivity in tumour and normal tissues to blood was determined. The tumour/blood ratio of the ^125^I-labelled A7-Ferumoxides increased in a time-dependent manner to 2.23±0.48 at 72 h after injection. By contrast, the tumour/blood ratio of ^125^I-labelled normal mouse IgG-Ferumoxides was lower than that of ^125^I-labelled A7-Ferumoxides (Figure 5).

### MR imaging of human nude mice bearing human colorectal carcinoma xenografts

**Figure 6:**
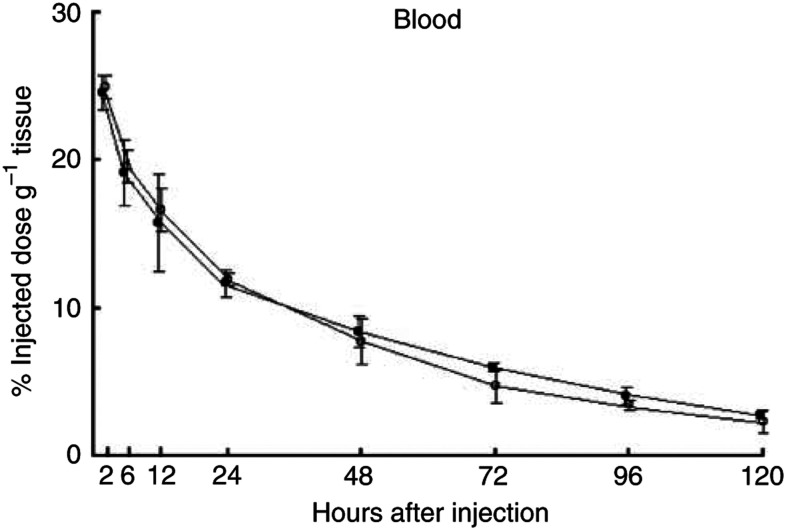
The signal intensity of MR T2-weighted imaging was reduced at the margin of tumours in an A7-Ferumoxides-injected mouse. By contrast, there was no change in intensity in tumours of a control IgG-Ferumoxides-injected mouse.

As shown in Figure 6, the signal intensity of MR T2-weighted imaging was reduced at the margin of tumours in a A7-Ferumoxides-injected mouse. By contrast, there was no change in intensity in tumours of a normal mouse IgG-Ferumoxides- injected mouse.

## DISCUSSION

Computed tomography and MR imaging are used currently for diagnosing postoperative local recurrence of rectal carcinoma. Although MR imaging is more successful than CT in the differentiation of local recurrence from postoperative fibrosis based on differences in signal intensity on T2-weighted images (Krestin *et al*, 1988), the diagnosis of local recurrence at early stages is often difficult. In many cases, tumours already have enlarged and invaded adjacent organs by the time of detection. Improvement of the prognosis might be expected from earlier diagnosis.

Recently, the clinical value of advanced imaging techniques for differentiation between local recurrence and other tissues has been evaluated. Positron emission tomography (PET) can complement MR imaging in the differential diagnosis between recurrent tumour and postoperative scar (Ito *et al*, 1992; Takeuchi *et al*, 1999). The values of CEA scintigraphy and dynamic MR imaging have also been evaluated (Muller-Schimpfle *et al*, 1993; Blomqvist *et al*, 1996; Jarv *et al*, 2000; Torricelli *et al*, 2003).

In the field of MR contrast agents, the usefulness of ferromagnetic colloidal particles coated with methacrylate copolymers coupled to antibodies has been reported (Renshaw *et al*, 1986a). Superparamagnetic iron oxide-based colloids as tissue-specific contrast agents have been developed. Superparamagnetic iron oxide produces dephased adjacent protons with loss of signal intensity in specific organs at low doses. Superparamagnetic iron oxide is magnetised within an external magnetic field and loses magnetisation without an external magnetic field. It disturbs a local magnetic field and shortens the transverse relaxation time. Ferumoxides and Ferucarbotran, which are SPIO-based colloids, are used clinically as liver-targeted MR contrast agents. They are trapped in phagocytic cells of the reticuloendothelial system and reduce the signal intensity of normal liver tissue in T2-weighted pulse sequences. As the signal intensity of the liver tumour without the reticuloendothelial system does not change, the contrast between liver tumours and normal liver tissues is improved. However, a contrast agent that is useful for local recurrence of rectal carcinoma has not been developed. We considered that development of an MR contrast agent that accumulates specifically in rectal carcinoma could make detection of local recurrence easier and lead to improvement of curability. We examined whether Mab A7 is applicable to an MR contrast agent for local recurrence of rectal carcinoma.

We have previously coupled Mab A7 with ferromagnetic particles (Bio-Mag4100) and reported its usefulness in rectal carcinoma (Kotani, 1995). However, it disappeared from the blood relatively slowly, and the tumour to blood ratio was not high enough to distinguish recurrent tumour reliably by MR imaging of patients (unpublished data). If SPIO is carried into tumour by monoclonal antibody, it is useful as an MR contrast agent for detection. We successfully coupled Mab A7 with Ferumoxides, and the immunoreactivity of the complex was retained. A7-Ferumoxides was stable in human serum for at least 5 days. Larger amounts of the complex accumulated in tumour than did normal mouse IgG-Ferumoxides. Furthermore, a larger amount of ^125^I-labelled A7-Ferumoxides accumulated in the tumour than in either blood or normal tissues. A7-Ferumoxides accumulated specifically in the tumour by antigen-antibody-specific binding. A7-Ferumoxides may therefore accumulate in recurrent tumours if administered to patients with local recurrence of rectal carcinoma. In MR imaging of nude mice bearing human colorectal carcinoma, the signal intensity was reduced at the margin of the tumour by injection of A7-Ferumoxides. The reason for the heterogeneity of localisation of A7-Ferumoxides in tumour may be explained by differences in blood supply within the tumour. In a previous study using ^131^I-labelled Mab A7, more antibody was localised at the margin than at the centre of a WiDr tumour by radioautography (unpublished data).

From these results, we conclude that Mab A7 coupled to Ferumoxides is potentially useful as an MR contrast agent for local recurrence of human rectal carcinoma.
